# Short-Term Rental Forecast of Urban Public Bicycle Based on the HOSVD-LSTM Model in Smart City

**DOI:** 10.3390/s20113072

**Published:** 2020-05-29

**Authors:** Dazhou Li, Chuan Lin, Wei Gao, Zihui Meng, Qi Song

**Affiliations:** 1 College of Computer Science and Technology, Shenyang University of Chemical Technology, Shenyang 110016, China; lidazhou@syuct.edu.cn (D.L.); centaureacyanus@foxmail.com (Z.M.); 2 Key Laboratory for Ubiquitous Network and Service Software of Liaoning province, Dalian University of Technology, Dalian 116024, China; chuanlin1988@gmail.com; 3 School of Computer Science and Engineering, Northeastern University, Shenyang 110819, China; songqi1002@163.com

**Keywords:** smart city, IoTs, meteorological, blocking, long short-term memory (LSTM), high order singular value decomposition (HOSVD), public bicycle system

## Abstract

As a kind of transportation in a smart city, urban public bicycles have been adopted by major cities and bear the heavy responsibility of the “last mile” of urban public transportation. At present, the main problem of the urban public bicycle system is that it is difficult for users to rent a bike during peak h, and real-time monitoring cannot be solved adequately. Therefore, predicting the demand for bicycles in a certain period and performing redistribution in advance is of great significance for solving the lag of bicycle system scheduling with the help of IoT. Based on the HOSVD-LSTM prediction model, a prediction model of urban public bicycles based on the hybrid model is proposed by transforming the source data (multiple time series) into a high-order tensor time series. Furthermore, it uses the tensor decomposition technology (HOSVD decomposition) to extract new features (kernel tenor) from higher-order tensors. At the same time, these kernel tenors are directly used to train tensor LSTM models to obtain new kernel tenors. The inverse tensor decomposition and high-dimensional, multidimensional, and tensor dimensionality reduction were introduced. The new kernel tenor obtains the predicted value of the source sequence. Then the bicycle rental amount is predicted.

## 1. Introduction

In November 2018 at the Foreign Relations Council in New York, Samuel Palmisano, CEO of IBM, put forward the concept of a “Smart Planet”. Intelligent technology is applied in many places in life. Transportation, electricity, food, currency, retail, infrastructure, and cities are becoming more and more intelligent, which has also made the earth continue to move towards the intelligent field. “Smart Earth” has stimulated the enthusiasm of countries to create smart cities.

Smart cities use information technology and other high-end technologies as the cornerstone, supported by the Internet of Things (IoT) [1,2,3] and cloud computing [4,5,6]. Taking intelligence, transparency, and networking as an essential means, on the one hand, it can reproduce the digital form of the material city. On the other hand, it can combine with the material city and derive a mutually beneficial urban system. Smart cities have significant ecological connotations and social responsibilities, embodying the duality of the integration of virtual and reality and the coexistence of advantages and disadvantages.

The smart city is the product of the new information technology revolution and the rapid development of society, culture, and economy, and it is a profound combination of the “three modernizations”. A variety of networks such as IoTs, telecommunication networks, Wi-Fi, and the internet are used as communication support layers. It is a new idea for urban development that takes the rapid development of smart industries, the deep integration of smart technologies, and the quick and convenient use of smart services as a benefit to the people.

The urban public bicycle system in the smart city powered by the IoTs is mainly composed of bicycle stations, parking piles, public bicycles, smart rental terminals, smart rental cards (generally bound to urban bus cards), control centers, vehicle dispatch centers, dispatch vehicles, and bicycle repair and maintenance bases. In the above links, a large amount of new intelligent computing algorithms will be needed in each link. For these enormous amounts of technical needs, the method of artificial intelligence (AI) must be used to realize the value of its application promptly [7,8,9].

In this paper, the LSTM prediction model is improved and a city public bicycle prediction model based on the hybrid model is proposed. Furthermore, a comprehensive forecasting model is established by comprehensively considering the effects of time and weather on public bicycle rental across the entire city. In the proposed model, we combine the low-rank tensor decomposition and the tensor prediction model into a unified framework, which is not only faster but also captures the internal relationship between multiple time series. It can improve prediction results, especially for short data. The proposed model is used to predict the number of rental public bicycles in the entire city. Finally, experimental prediction and analysis are performed to obtain the prediction results of the model.

## 2. Related Works

Many scholars have studied the relevant theories of urban public bicycle systems. Singhvi et al. predicted pairwise bike demand for New York City’s Citi Bike system [10]. Kou et al. analyzed the distributions of trip distance and trip duration for bike-sharing trips for commuting and touristic purposes [11]. Raviv and Kolka introduced an inventory model suited for the management of bike rental stations and a numerical solution method used to solve it. Moreover, a structural result about the convexity of the model has been proved [12]. Mauro et al. addressed the Bike-Sharing Rebalancing Problem (BRP), in which a fleet of capacitated vehicles is employed to redistribute the bikes to minimize total cost [13]. Qiu and He comprehensively analyzed the domestic urban bicycle system and studied the relationship between economic development and transportation development [14].

Kloimüllner and Raidl investigated a simplified problem model in which only full vehicle loads had been considered for movement among the rental stations. It appears to have only a minor impact on the achieved quality of the rebalancing in practice but eases the modeling substantially. More specifically, the rebalancing problem as a selective unit-capacity pickup and delivery problem with time budgets on a bipartite graph has been formulated, and a compact mixed-integer linear programming model, a logic-based Benders decomposition and a variant thereof, namely branch-and-check for it, has been given [15]. Caggiani et al. presented a multi-objective model based on a fuzzy inference system to be embedded in a mobile application that could assist cyclists in the selection of the smartest route to follow to reach their destination in terms of travel costs (distance or time), level of air pollution and road safety [16]. Pal and Zhang presented a Novel Mixed Integer Linear Program for solving the Static Complete Rebalancing Problem. The proposed formulation can not only handle single as well as multiple vehicles but also allows for multiple visits to a node by the same vehicle [17]. Zhao et al. studied the dispatching and management of no pile bicycle-sharing and constructed a dispatching model based on semi-open multi-vehicles with fuzzy time windows, with the number of distribution vehicles and dispatching cost as the objective function [18]. Ho and Szeto proposed a hybrid massive neighborhood search for solving the problem. Several removals and insertion operators have been proposed to diversify and intensify the search [19]. Goh et al. proposed a method for estimating the primary demand using a rank-based demand model which accounts for choice substitutions by treating each observed trip as the best available option in a latent ranking over origin-destination (OD) pairs [20]. Yang et al. proposed a spatiotemporal bicycle mobility model based on historical bike-sharing data and devised a traffic prediction mechanism on a per-station basis with sub-hour granularity [8]. Liu et al. proposed an inter-station bike transition (ISBT) model to predict the station drop-off demand. Moreover, a mixed-integer nonlinear programming (MINLP) formulation of multiple capacitated bike routing problems to minimize total travel distance has been provided [21].

LSTM model, also known as the long- and short-time memory model, is a kind of RNN, but also, by the input layer, hidden layer, and output layer, is a new kind of deep machine learning neural network. LSTM and traditional RNN neural networks are similar. The difference is that LSTM with a memory module instead of an RNN neural network has an implicit layer node so that it has memory ability. Ai et al. employed a deep learning approach, named the convolutional long short-term memory network (Conv-LSTM), to address the spatial dependencies and temporal dependences [22]. Xu et al. developed the LSTM model to predict the bike-sharing trip production and attraction for different time intervals, including for 10-min, 15-min, 20- min, and 30-min intervals [23]. Zhang et al. considered both historical usage and real-time passengers of public transport and used the LSTM model to establish the connection among them [24]. Pan et al. proposed a real-time LSTM model for predicting bike demands in different areas of a city during a future period based on historical data from Citi Bike System Data and meteorology data [25]. Wang et al. focused on the short-term forecasting for docking station usage in the case of Suzhou, China [26]. In [26], two latest and highly efficient models, LSTM and GRU, are adopted to predict the short-term available number of bikes in docking stations with one-month historical data.

## 3. Method

**Figure 1:**
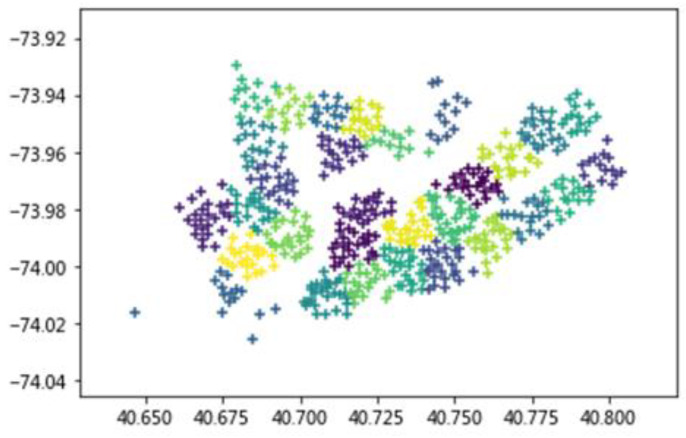
The stations in the geographic coordinates.

Each station in the New York City public bicycle trip data published on the Public Bicycle System website corresponds to a unique ID number. As the station name is relatively long, this section will use the corresponding ID number to indicate the station. Figure 1 shows the stations in the geographic coordinates. Different colors represent different blocks. There are 30 blocks in total. Among them, the abscissa is the latitude of the bicycle station and the ordinate is the longitude of the bicycle station.

As shown in Table 1, Block 1 contains nine bicycle stations. Block 2 contains 12 bicycle stations. Block 3 contains ten bicycle stations. Block 4 contains two bicycle stations, which is also the block with the least number of stations because it is relatively remote and there are only two isolated stations around it. Block 8 contains 15 bicycle stations, which is also the block with the most stations since it is located in the center of New York and the site is more concentrated.

The traditional algorithm uses only the trip data of the predicted bike-sharing station to predict the number of rentals and returns to that bike-sharing station in the future. The bike-sharing station as the predicted object is viewed separately and in isolation from other bike-sharing stations during the prediction process. However, there is a strong correlation between one bike-sharing station and other bike-sharing stations in the same time frame. If this strong correlation is overlooked in the prediction process, then the accuracy of the calculation results is bound to suffer. Besides, in large cities with many bike-sharing stations, the number of rentals and returns for each bike-sharing station is predicted individually, which also reduces computational efficiency and extends the computational time for the whole city.

To overcome the above problems of the traditional algorithm, we used the whole public bicycle trip data of all bike-sharing stations in the city as the prediction object. The trip data for all bike-sharing stations throughout the city can be introduced in one prediction process. The method not only introduces the relevant effects between all bike-sharing stations at the same time into the calculation but also avoids the inefficient calculations that result from predicting each bike-sharing station individually. We summarize some notations used in our task definition in Table 2.

As described in Table 1, there are 74 bike-sharing stations in the area. Each bike-sharing station generates two sets of public bicycle trip data every hour. These two records respectively record the number of public bicycles rented and the number of public bicycles returned within 1 h. If the recording time is set to 5000 h, then the total number of public bicycle rentals and repayments of all 74 bike-sharing stations within 5000 h will constitute a high-level tensor. The dimension of this tensor is 74 × 5000 × 2, and we use the symbol ***M*** to represent this tensor. The dimension of ***M*** is equal to $I_{1}\;$×$\;I_{2}\;$× $I_{3}$ in the data set scenario described above, where $I_{1}\;$= 74, $I_{2}\;$= 500, and $I_{3}\;$= 2.

We applied HOSVD’s decomposition of a higher-order tensor $M\in R^{I_{1}\times I_{2}\times I_{3}}$ into a kernel tensor $G\in R^{R_{1}\times R_{2}\times R_{3}}$ and a series of factor submatrixes $U_{1},U_{2},U_{3}$ of the modular-*3* product.

(1) $$M=G\times_{1}U_{1}\times_{2}U_{2}\times_{3}U_{3}$$

Among them, $U_{i}\in R^{I_{i}\times R_{i}},\;i=1,\;2,\;3$ is orthogonal. Generally, $R_{i},\;i=1,\;2,\;3$, the scale of the kernel tensor G obtained by decomposition, is smaller than $I_{3},\;i=1,\;2,\;3$. When we set $I_{3}$ = 2 to present the range of the public bicycle trip data as the rentals and returns, we can obtain $R_{3}=1$ for $R_{3}<I_{3}$. The kernel tensor $G\in R^{R_{1}\times R_{2}\times 1}$ is a matrix. In this sense, the storage in HOSVD’s decomposition is much smaller than the storage of the original tensor. Therefore, we are particularly interested in extending the HOSVD decomposition for tensor analysis.

For a given tensor ***M***, the goal is to solve a low-rank tensor $X\in R^{I_{1}\times I_{2}\times I_{3}}$ to get the following low-rank tensor problem:

(2) $$min_{X}\frac{1}{2}\|X-M{\|}_{F}^{2}$$

where ${\left\|\;\right\|}_{F}^{2}$ denotes the second order Frobenius norm.

Unlike the matrix case, the low-rank tensor estimation problem defined by Equation (2) is usually difficult to solve [27]. Inspired by the sparse representation, it is transformed and replaced by a (weighted) trace norm minimization problem:

(3) $$min_{X}\overset{3}{\underset{i=1}{\sum }}\|X_{\left(i\right)}{\|}_{tr}+\frac{\lambda }{2}\|X-M{\|}_{F}^{2}$$

where $X_{\left(i\right)},\;i=1,2,3$ is the module-*i* expansion matrix of ***X***, $\|X_{\left(i\right)}{\|}_{tr}$ is the trace norm of the matrix $X_{\left(i\right)}$ which is the sum of singular values of $X_{\left(i\right)}$, and λ > 0 is the regularization parameter.

Some scholars [28,29] have provided some matrix rank estimation methods to calculate some values of the three ranks of the tensor involved. Therefore, we consider some integers $R_{i},\;i=1,\;2,\;3$.

In [30], let tensor $X\in R^{I_{1}\times I_{2}\times I_{3}}$ and $G\in R^{R_{1}\times R_{2}\times R_{3}}$ satisfy $X=G\times_{1}U_{1}\times_{2}U_{2}\times_{3}U_{3}$, and $U_{i}^{T}U_{i}=E_{R_{i}},\;i=1,\;2,\;3$, *T* indicate matrix transposition. $E_{R_{i}}$ is a $R_{i}$-dimensional unit matrix, and then

(4) $$\|X_{\left(i\right)}{\|}_{tr}=\|G_{\left(i\right)}{\|}_{tr},\;i=1,\;2,\;3$$

The module-*i* expansion of matrix 1***X*** and ***G*** are $X_{\left(i\right)}$ and $G_{\left(i\right)}$, respectively. Based on Equation (4), Equation (3) can be transformed into

(5) $$\begin{array}{c} min_{G,\left\{U_{i}\right\}}\overset{3}{\underset{i=1}{\sum }}\|G_{\left(i\right)}{\|}_{tr}+\frac{\lambda }{2}\|M-G\times_{1}U_{1}\times_{2}U_{2}\times_{3}U_{3}{\|}_{F}^{2}\\ s.t.\;\;U_{i}^{T}U_{i}=E_{R_{i}}\;,\;i=1,\;2,\;3 \end{array}$$

The kernel tensor trace norm is Equation (5), which can alleviate the large expansion matrix involved in the convex decomposition problem and SVD computational burden. Besides, the trace norm regularization term in Equation (5) is used to improve the robustness of rank selection. The original Higher-Order Singular Value Decomposition (HOSVD) method is usually for a given rank is very sensitive [31]. Due to the interdependent matrix trace norm term, auxiliary variable $G_{i}\in R^{R_{i}\times \prod_{j\neq i}R_{j}}$ is introduced into Equation (5), and Equation (5) is re-represented as the following equivalence:

(6) $$\begin{array}{c} min_{G,\left\{U_{i}\right\}}\overset{3}{\underset{i=1}{\sum }}\|G_{\left(i\right)}{\|}_{tr}+\frac{\lambda }{2}\|M-G\times_{1}U_{1}\times_{2}U_{2}\times_{3}U_{3}{\|}_{F}^{2}\\ s.t.\;\;G_{i}=G_{\left(i\right)},\;U_{i}^{T}U_{i}=E_{R_{i}}\;,\;i=1,\;2,\;3 \end{array}$$

The Lagrangian function of Equation (6) is

(7) $$\begin{array}{cc} L & =\left(G,U_{1},U_{2},U_{3},G_{1},G_{2},G_{3},Y_{1},Y_{2},Y_{3}\right)\\ & =\overset{3}{\underset{i=1}{\sum }}\|G_{i}{\|}_{tr}+\frac{\lambda }{2}\|M-G\times_{1}U_{1}\times_{2}U_{2}\times_{3}U_{3}{\|}_{F}^{2}+\overset{3}{\underset{i=1}{\sum }}\frac{\mu }{2}\|G_{\left(i\right)}-G_{i}+{\frac{Y_{i}^{}}{\mu }\|}_{F}^{2}-\overset{3}{\underset{i=1}{\sum }}\frac{\mu }{2}\|\frac{Y_{i}^{}}{\mu }\| \end{array}$$

where ‖ ‖ is the norm, μ > 0 is a regularization parameter, and the multiplier variable is $Y_{i}^{}$.

Next, we use an iteration algorithm to solve Equation (7). With the calculation process in its *k*-th iteration, the calculation of $G^{k+1}$, $U_{1}^{k+1},U_{2}^{k+1},U_{3}^{k+1}$ is the object. For $G^{k+1}$, we add neighbor operator $\frac{\tau }{2}\|G_{}-G_{}^{k}{\|}_{F}^{2}$, τ > 0 as a regularization parameter, and we obtain

(8) $$\begin{array}{c} G^{k+1}=min_{G}\frac{\lambda }{2}\|M-G\times_{1}U_{1}\times_{2}U_{2}\times_{3}U_{3}{}_{F}^{2}+\overset{3}{\underset{i=1}{\sum }}\frac{\mu }{2}\|G_{\left(n\right)}-G_{n}^{k}+{\frac{Y_{n}^{k}}{\mu }\|}_{F}^{2}+\frac{\tau }{2}\|G-G_{}^{k}{\|}_{F}^{2} \end{array}$$

For $U_{1}^{k+1},U_{2}^{k+1},U_{3}^{k+1}$, $U_{i},\;i=1,\;2,\;3\;$ can be solved by fixing other variables $U_{j}$, *j* ≠ *i*, j=1, 2, 3. Equation (5) is transmitted to the following problems:

(9) $$\begin{array}{c} min_{U_{i}}\|M-G^{k+1}\times_{1}U_{1}^{k}\cdots \times_{i-1}U_{i-1}^{k}\times_{i}U_{i}^{k}\times_{i+1}U_{i+1}^{k}\cdots \times_{3}U_{3}^{k}{\|}_{F}^{2}\\ s.t.\;\;U_{i}^{T}U_{i}=E_{R_{i}}\;,\;i=1,\;2,\;3 \end{array}$$

Considering that the matrix $U_{i}$ is column orthogonal, we can get

(10) $$U_{i}^{k+1}=\hat{U_{n}}\hat{V_{n}}$$

In Equation (10), $\hat{U_{n}}$ and $\hat{V_{n}}$ are given by the singular value decomposition of Equation (9).

For $G_{i}^{k+1}$, i=1, 2, 3,  we add neighbor operator $\frac{\tau_{i}}{2}G_{i}-G_{i}^{k}{}_{F}^{2}\;$ and we obtain

(11) $$\begin{array}{c} G_{i}^{k+1}=min_{\;G_{i}}\|G_{\left(i\right)}{\|}_{tr}+\frac{\mu }{2}\|G_{\left(i\right)}^{k}-G_{\left(i\right)}+{\frac{Y_{i}^{k}}{\mu }\|}_{F}^{2}+\frac{\tau_{i}}{2}\|G_{i}-G_{i}^{k}{\|}_{F}^{2} \end{array}$$

According to [32], $G_{i}^{k+1}$ can be obtained from Equation (11)

(12) $$G_{i}^{k+1}=D_{\frac{1}{\mu +\tau_{i}}}\left(\frac{\mu G_{\left(i\right)}^{k}+Y_{i}^{k}+\tau_{i}G_{i}^{k}}{\mu +\tau_{n}}\right)$$

For the $Y_{i}^{k+1},\;i=1,\;2,\;3,$ multiplier variable, it can be calculated as follows

(13) $$Y_{i}^{k+1}=Y_{i}^{k}+\mu \gamma \left(G_{\left(i\right)}^{k+1}-G_{i}^{k+1}\right)$$

Taking the data set scenario described in Table 1 as an example, all 74 bike-sharing stations within 5000 h will constitute a high-level tensor $M\in R^{I_{1}\times I_{2}\times I_{3}},\;I_{1}=74,I_{2}=5000,I_{3}=2$, which is the input of the method. After processing through the HOSVD decomposition algorithm, the kernel tensor $G\in R^{R_{1}\times R_{2}\times R_{3}}$ can be obtained since $R_{3}<I_{3}$ and $I_{3}=2$, $R_{3}=1$ is the only result. In Table 3, we can set $R_{1}$ or $R_{2}$ to one at the start of the HOSVD decomposition algorithm. Finally, the kernel tensor G can be obtained as a vector, because two of the three-dimensionality equal one.

When the kernel tensor G is a vector, we can forecast the new G′ with the LSTM model with the historical G. The new G′ can be used to multiply the matrixes $U_{1},U_{2},U_{3}$, which are also the result of the HOSVD decomposition algorithm, as listed in Table 3. According to Equation (1), the prediction of high-order tensor M′ equals the result of $G\prime \times_{1}U_{1}\times_{2}U_{2}\times_{3}U_{3}$. Because the prediction of M′ is the whole public bicycle trip data of all bike-sharing stations in the city in the next hour, we only have to use the LSTM method once.

LSTM in the sequence of data prediction has a good effect at present in the stock price prediction, and other fields have been widely used. Existing algorithms mostly use LSTM to predict the number of public bicycles to be leased and returned at the next moment of a bike-sharing station. The algorithm in this paper then uses LSTM to predict the kernel tensor G′, which is also an eigenvector of the whole public bicycle trip data of all bike-sharing stations in the city in the next moment.

We built an LSTM model with one input layer, three hidden layers, and one output layer to predict the new ***G′*** vector at the next moment, where ***G*** is the input vector of the current moment, and ***G′*** is the output value obtained after training. The model propagation process is trained according to the backpropagation through the time algorithm. The predicted value of the final moment is obtained when the error is minimal, or the maximum number of iterations is reached. The theoretical content and detail of the LSTM are available in [23].

We built the model based on the deep learning framework TensorFlow. The activation function is set to tanh, the number of cycles is 30, the number of time steps is 24, the number of training samples in each batch is 16, the number of neurons in the hidden layers is 20, and the learning rate is 0.001. In TensorFlow, the processing steps of the LSTM model are shown as follows.

Step 1: Prepare the vector G as a sequence number, which is the output of the HOSVD decomposition algorithm listed in Table 3;
Step 2: Determine the structure of the LSTM, such as the number of hidden layers and the neurons in each layer;
Step 3: Choose the right activation function and the right optimization algorithm;
Step 4: Use the training set data to train and optimize the neural network to obtain the prediction model;
Step 5: Use the validation data set to validate the model prediction effect. If the effect is good, continue to predict, then go to Step 6, otherwise return to Step 2;
Step 6: Use the model with good prediction results and test data to predict ***G′*** in the future.

When we have the vector ***G′*** through the LSTM model, the prediction of the whole public bicycle trip data of the all bike-sharing stations in the city in the next moment, M′, equals the result of $G\prime \times_{1}U_{1}\times_{2}U_{2}\times_{3}U_{3}$, $U_{1},U_{2},U_{3}$ and are also the output of the HOSVD decomposition algorithm, as listed in Table 3.

## 4. Experiment and Result Analysis

### 4.1. Dataset

(1) The first step was to sort the prediction block data. Block 8 included 15 sites with site IDs 3254, 2008, 534, 427, 415, 376, 360, 351, 337, 315, 304, 264, 260, 259, and 195. During the period from 10:00 to 11:00 on 11 September 2016, the number of bicycles rented out at the above eight stations was 4, 11, 20, 9, 17, 6, 4, 1, 0, 6, 13, 9, 9, 4 and 0, respectively. The number of bicycles rented out by Block 8 during this period is the sum of the rental amounts of the above stations, which is 113 bikes. Taking the data interval as 1 h; the bicycle rental amounts of Block 7 and Block 8 in each period were calculated as the following experimental data.
(2) The second step was to select data for training the model. Based on the experimental data compiled in the first step, the bicycle rental data of bicycle stations from 9 August 2016, to 20 September 2016 were used as training data for this experiment. Since the format of the time record in the original data was 9/11/2016 00:00:06, to facilitate training we used spaces instead of ‘/,’ and then entered it in the table.
(3) The third step was to select real data for comparison with predicted data. Based on the experimental data compiled in the first step, the bicycle rental data from September 21 to 30 September 2016were used as test data for this experiment. The model input data processing is the same as in the second step.

### 4.2. Result Analysis

**Figure 2:**
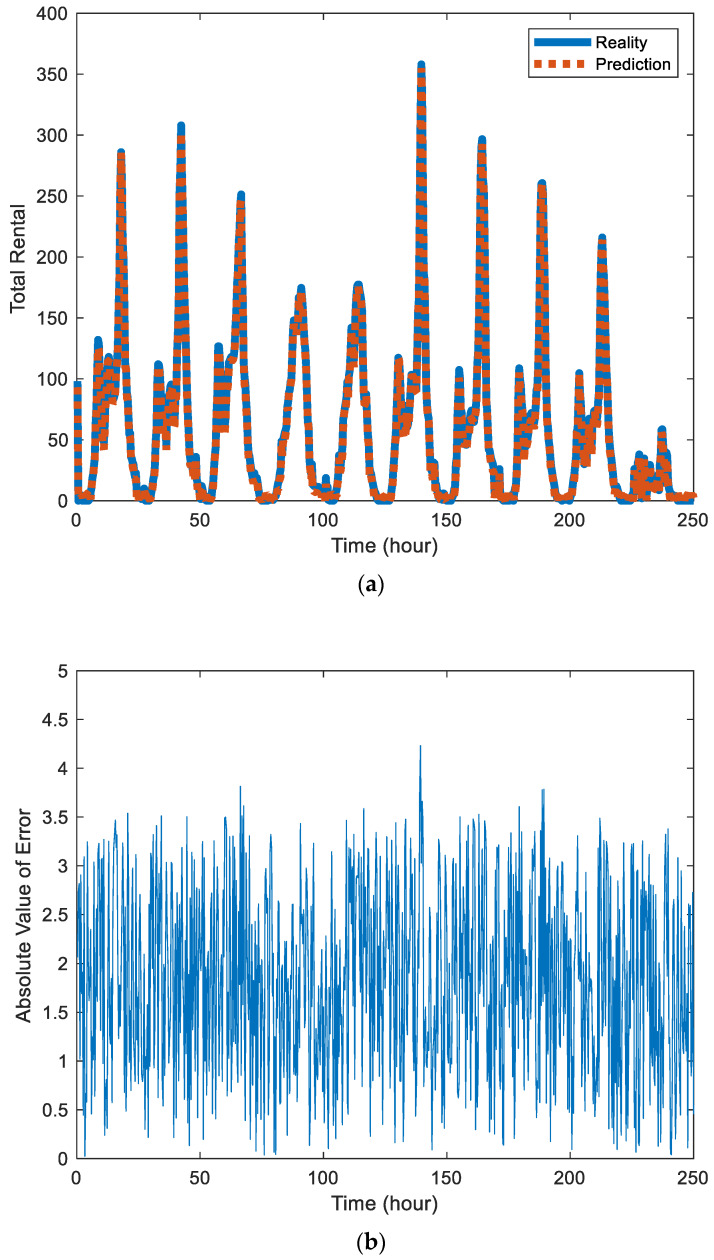
(**a**) Comparison chart of the predicted value and actual value of public bicycle rental in station Block 7. (**b**) Absolute value of error between the predicted value and actual value in the station Block 7.

**Figure 3:**
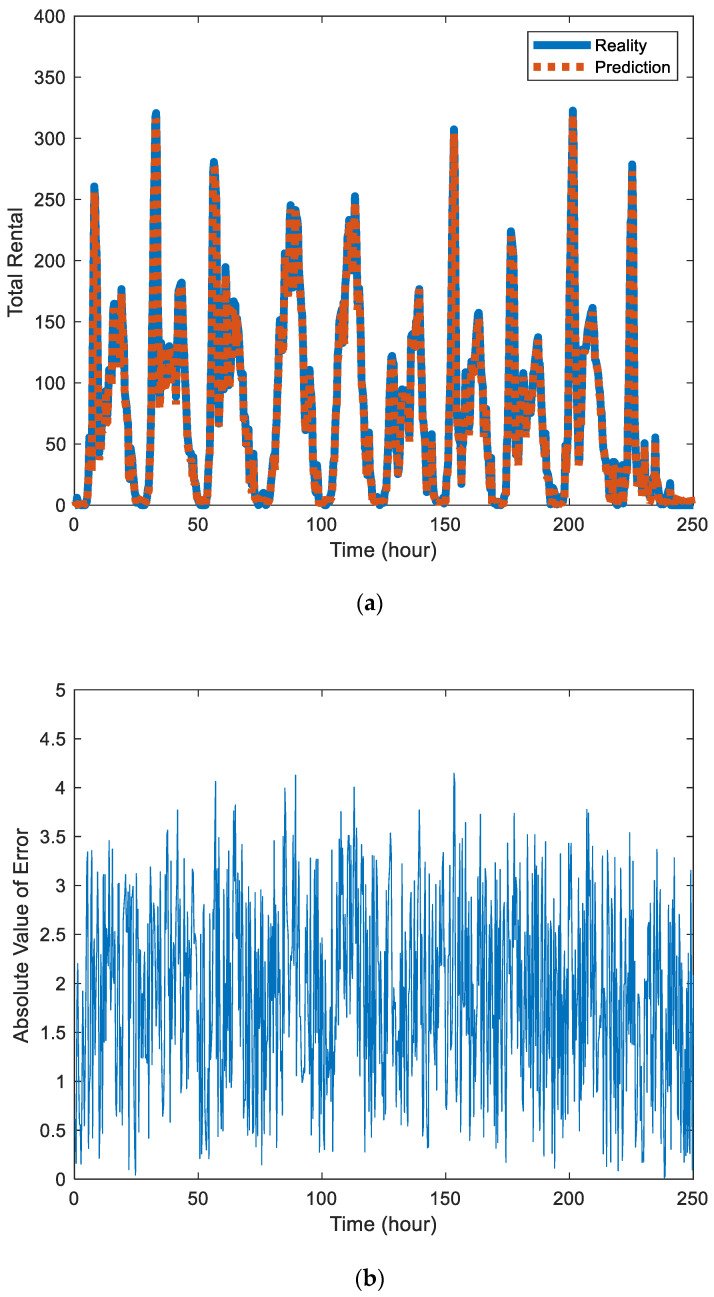
(**a**) Comparison chart of the predicted value and actual value of public bicycle rental in station Block 8. (**b**) Absolute value of error between the predicted value and actual value in the station Block 8.

Figure 2a and Figure 3a are the prediction results of Block 7 and Block 8, where the solid blue line represents the real value and the red dotted line represents the prediction. By comparison, it can be found that the prediction effect of Block 7 is better than that of Block 8 because Block 7 contains fewer sites, has a small number of users per day, is relatively stable, and is easier to predict.

Figure 2b and Figure 3b are the absolute values of error between the predicted value and actual value in the station Block 7 and Block 8, respectively. The error data also contains certain components of periodicity. There are also differences in the distribution of errors in different urban areas.

**Figure 4:**
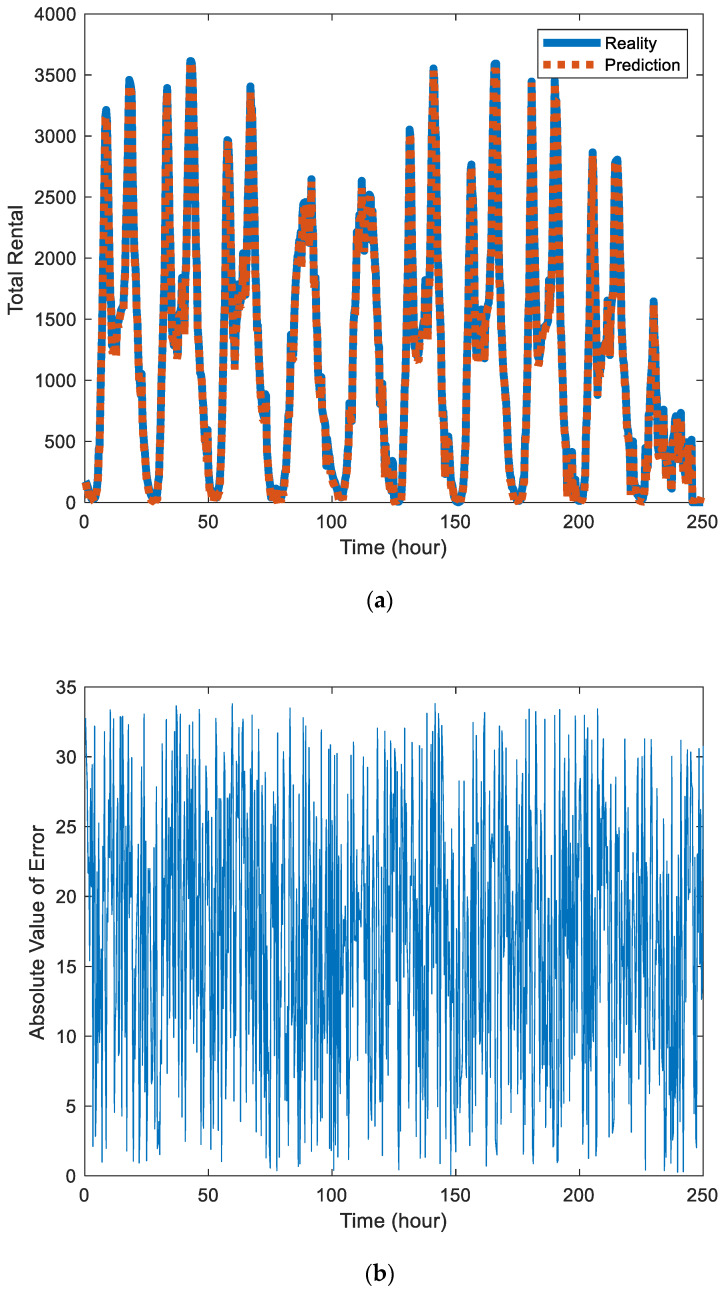
(**a**) Comparison of predicted value and actual value of public bicycle rental in the whole city. (**b**) The absolute value of error between the predicted value and actual value in the whole city.

Figure 4a is a comparison chart of the public bicycle rental predicted by the proposed model with the real value in the whole city. Figure 4b is the absolute value of error between the predicted value and actual value in the whole city. In the error data, periodicity still exists but the scope of review increases.

The indicators used to measure the results between predicted values and reality are Root Mean Squared Logarithmic Error (RMLSE) and error rate (ER).

(1) As shown in Table 4, the mean, data amount N, standard deviation, and standard error of the mean are given. The standard deviations of the true and predicted values are 68.753, 67.755, 25.520, and 26.789, respectively. The true and predicted values of public bicycle rental differ greatly from their respective averages, indicating the instability of public bicycle rental.
(2) As shown in Table 5, the correlation coefficient and significance of the number of cases between the true value and the predicted value are given. The correlation coefficient indicates the strength of the correlation between the true value and the predicted value. The closer the absolute value is to 1, the stronger the correlation. Generally, when the absolute value is high and 0.75, the two sets of data have a strong correlation. From Table 5, it can be seen that the correlation coefficient distribution of the two groups of data is 0.956 and 0.929, which is much higher than 0.75, so the true value has a high correlation with the predicted value. When the significance is less than 0.05, the two sets of data are statistically significant, and the annotation shows that the parameter is 0.000 so the true and predicted values are statistically significant.
(3) Table 6 shows a 95% confidence interval of the difference between the real and predicted values.
(4) Root Mean Squared Logarithmic Error (RMLSE) and Error Rate (ER).
  (14) $$RMLSE=\frac{1}{T}\sum_{t=1}^{T}\sqrt{\frac{1}{m}\sum_{i=1}^{m}(\log(\hat{XCi,t}+1)-\log(XCi,t}{+1))}^{2}$$
  (15) $$ER=\frac{1}{T}\sum_{t=1}^{T}\frac{\sum_{i=1}^{m}\left|\hat{XCi,t}-XCi,t\right|}{\sum_{i=1}^{m}XCi,t}$$

Here XCi,t is the real value of the car rental in the Block Ci during the period t and $\hat{XCi,t}$ is the corresponding predicted value. Table 7 shows the root mean square error and error rate of the Block rental.

Table 8 shows RMLSE and ER of the proposed HOSVD-LSTM method, the classic LSTM in [23,24,25,26], and the CNN-LSTM in [22] in the same experimental setting. The HOSVD-LSTM has a relatively 3.551 and 1.459 lower RMLSE and 2.623 and 0.851 lower ER than the classic LSTM in [23,24,25,26] and the CNN-LSTM in [22], demonstrating that HOSVD-LSTM performs better in time series prediction.

## 5. Discussion

### 5.1. The Applied Method in a Real Scenario with Real Data

**Figure 5:**
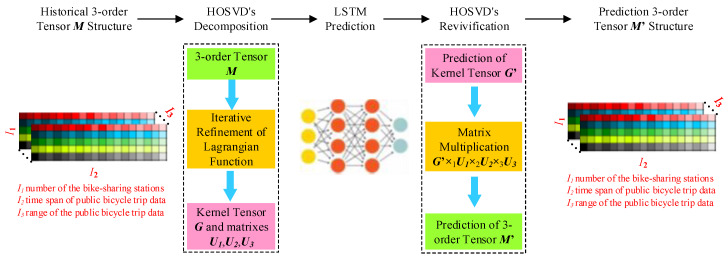
The processing flow of prediction in a real scenario with real data.

Figure 5 shows the flow of prediction in a real scenario with real data. The input of the HOSVD-LSTM model is whole public bicycle trip data of all bike-sharing stations in the city, ***M***, which is a tensor with the dimension is *I*_1_×*I*_2_×*I*_3_. *I*_1_ is the number of bike-sharing stations. *I*_2_ is the period of the public bicycle trip data. *I*_3_ is the range of public bicycle trip data. The 3-order tensor ***M*** can be decomposed by the iterative refinement in the Lagrangian function of HOSVD method to the kernel tensor G and a series of factor submatrixes $U_{i}\in R^{I_{i}\times R_{i}},\;i=1,2,3$. When the kernel tensor G is a vector, we can forecast the new G′ in the LSTM prediction step in Figure 5 with the historical G. In the HOSVD’s revivification step in Figure 5, G′, the result of LSTM prediction, and $U_{i}\in R^{I_{i}\times R_{i}},\;i=1,2,3$, the result of HOSVD’s decomposition, can be used to obtain the prediction of 3-order tensor M′ according to Equation (1) in which $M^{\prime }=G\prime \times_{1}U_{1}\times_{2}U_{2}\times_{3}U_{3}$. The distribution of public bicycle trip data within one hour of the all bike-sharing stations in the area can be extracted from the $M^{\prime }$, which is the result of the HOSVD-LSTM model, as demonstrated in Figure 5.

### 5.2. Comparison with Existing Methods

In [22,23,24,25,26], the LSTM model was used in forecasting the urban public bicycle. In this section, we can compare the proposed HOSVD-LSTM model with [22,23,24,25,26] in the algorithm structure and the real data experiment.

In [23,24,25,26], the LSTM models were classic models used to predict the bike-sharing trip production. The predicting procedure begins with standardizing the historical data [23]. The observations of the selected variables were then used as inputs of the developed LSTM [23]. After the predicted future production, the mean absolute percentage error was used to compare the predicted results with the original production and attraction data [23].

However, the classic LSTM model cannot capture the spatial and temporal dependences simultaneously. Because it fails to capture the spatial dependencies, the classic LSTM is not an ideal model for the spatiotemporal distribution of sharing bikes. To overcome the above problem, we applied the spatiotemporal variables as spatiotemporal 3D tensors within the LSTM model in [22]. The CNN model was used to combine the 3D tensors in [22].

The proposed method in this paper has the same features as in [22]. The proposed method and [22] both take into account the spatiotemporal correlation of the data distribution. The proposed method used the HOSVD to simultaneously predict the public bicycle trip data for all bike-sharing stations in the entire city as a whole. Thus, the proposed method in this paper can consider the spatiotemporal dependencies, which have been neglected by the classic LSTM model.

Moreover, the result of HOSVD is also an eigenvector of the whole public bicycle trip data of all bike-sharing stations in the city in the next moment. The result of HOSVD has actual spatiotemporal characteristic information because HOSVD is an effective method of tensor decomposition and dimensionality reduction. The HOSVD is a widely used algorithm in machine learning, not only for feature decomposition in downscaled algorithms, but also for recommendation systems and natural language processing, among others. The HOSVD can not only decompose high-dimensional tensor but also reconstruct the decomposed high-dimensional tensor. Compared with the CNN used in [22], the HOSVD has more interpretability and more reliable reduction. The key difference is that the CNN result is an artificial approximation of the optimal spatiotemporal feature, whereas the HOSVD result is essential and mathematically optimal. As shown in Table 8, the proposed HOSVD-LSTM has better experimental results than the classic LSTM in [23,24,25,26] and the CNN-LSTM in [22] under the same experimental scenario.

## 6. Conclusions

We used the publicly available dataset of New York Bike for AI modeling, and the model outputs the predicted value of bicycle rental in the next hour and compares it with the actual value. Based on the extension of the HOSVD-LSTM model, a hybrid model was established to predict the rental of urban public bicycle station stations. This article combines the advantages of both low-rank tensor decomposition technology and the tensor prediction model into a unified framework. It is not only faster but also captures the internal relationship between multiple time series, which can improve prediction results, especially for short data. Next, we verified the actual effect of HOSVD-LSTM through a large number of experiments.

## Figures and Tables

**Table 1:** Block number and site ID number.

Block Number	Site ID Number
1	3430 3094 3089 3086 3084 3083 3081 3076 3074
2	3429 3417 3415 3413 467 416 353 298 274 243 241 83
3	3224 434 405 358 346 284 247 238 225 212
4	3182 3036
5	3427 3263 3260 3244 383 382 380 369 368 357 348
6	336 335 303 280 254 253 252 229 161 151 128
7	3423 3310 3306 3300
8	3254 2008 534 427 415 376 360 351 337 315 304 264 260 259 195

**Table 2:** The symbols used in the proposed method.

Symbol	Meaning of Symbolic Representation
***M***	whole public bicycle trip data of all bike-sharing stations in the city, ***M*** is a tensor, the dimension is *I*_1_ × *I*_2_ × *I*_3_
*I*_1_	number of the bike-sharing stations
*I*_2_	the period of the public bicycle trip data
*I*_3_	range of the public bicycle trip data
G	kernel tensor $G\in R^{R_{1}\times R_{2}\times R_{3}}$
$U_{1},U_{2},U_{3}$	a series of factor submatrixes from the HOSVD decomposition method, $U_{i}\in R^{I_{i}\times R_{i}},\;i=1,2,3$
X	a low-rank tensor $X\in R^{I_{1}\times I_{2}\times I_{3}}$
$X_{\left(i\right)}$	$X_{\left(i\right)},\;i=1,2,3$ is the module-*i* expansion matrix of ***X***
λ, μ,τ	λ > 0, μ > 0, τ > 0, regularization parameters
$E_{R_{i}}$	$R_{i}$-dimensional unit matrix, i=1,2,3
$G_{\left(i\right)}$	the module-*i* expansion matrix of ***G***, i=1,2,3
$Y_{i}^{}$	a multiplier variable
*k*	*k*-th iteration
$\hat{U_{n}}\hat{,V_{n}}$	results of singular value decomposition

**Table 3:** Higher-Order Singular Value Decomposition (HOSVD) algorithm detailed process description.

**Input: Given the Tensor *M*, the Scale of The Kernel Tensor**$R_{1},R_{2},R_{3}$, **Parameter**λ** = 100.** **Output: Kernel Tensor *G*, Matrixes**$U_{1},U_{2},U_{3}$
Initialization: $Y_{i}^{0}=0,\;G_{i}^{0}=0,\;U_{i}^{0}=rand\left(I_{i},R_{i}\right),\;\mu^{0}=10^{-4},\mu_{max}=10^{10}$
Step 1: Calculate $G^{k+1}$ the tensor according to Equation (8).
Step 2: For i=1,2,3, calculate $\mathrm{the}\;\mathrm{tensor}\;U_{i}^{k+1},\;G_{i}^{k+1}\;and\;Y_{i}^{k+1}$ according to Equations (11)–(13).
Step 3: Calculation parameter $\mu^{k+1}=min\left(\lambda \mu^{k},\mu_{max}\right)$
Step 4: Determine the convergence condition $max_{i=1,2,3}\left(G_{i}^{k+1}-G_{i}^{k}{}_{F}^{2}\right)<0.001$, if it meets, stop; otherwise, go to Step 1.

**Table 4:** Paired sample statics.

	Mean	N	Standard Deviation	the Standard Error of the Mean
Block 7 reality	60.72	240	68.753	4.447
Block 7 prediction	62.52	240	67.755	4.383
Block 8 reality	25.65	240	25.520	1.647
Block 8 prediction	27.55	240	26.789	1.729

**Table 5:** Correlation coefficients of the paired sample.

	N	Correlation Coefficient	Sig
Block 7 reality vs prediction	240	0.956	0.000
Block 8 reality vs prediction	240	0.929	0.000

**Table 6:** Test of the paired sample.

	Block Seven Reality vs. Prediction	Block Eight Reality vs. Prediction
95% confidence upper	−3.695	−2.980
95% confidence lower	0.097	−0.829
t	−1.869	−3.488
degrees of freedom	239	239
Sig (both sides)	0.063	0.001

**Table 7:** The error of rental.

	RMLSE	ER
*Block 7*	0.276	0.270
*Block 8*	0.320	0.322

**Table 8:** Comparison with existing methods.

	RMLSE	ER
*HOSVD-LSTM*	0.395	0.401
*classic LSTM*	3.946	3.024
*CNN-LSTM*	1.854	1.252
